# A positive focus on the mental health of parents

**DOI:** 10.2471/BLT.25.294704

**Published:** 2026-03-04

**Authors:** Indrani Paul, Richard Appiah, Nitika Pant Pai, Marilyn N Ahun

**Affiliations:** a Department of Medicine, Faculty of Medicine and Health Sciences, McGill University 5252 Boulevard de Maisonneuve Ouest, Office 2B.37, Montréal QC, H4A 3S5, Canada.; b College of Health Sciences, University of Ghana, Accra, Ghana.

The transition to parenthood, which spans pregnancy to the first few years of life, is a period of heightened vulnerability, as parents face increased caregiving demands, disrupted sleep, and economic and social pressures that can elevate the risk of mental health difficulties and compromise parental well-being.^1^ Importantly, poor parental mental health has consequences that extend beyond parents themselves, influencing children’s emotional, behavioural and developmental outcomes and reinforcing intergenerational patterns of vulnerability.^2^^,^^3^ When parental mental health difficulties go unrecognized or untreated, their effects could reverberate across generations, shaping children’s emotional regulation, behavioural development and long-term mental health trajectories.^2^^,^^3^ Research on parental mental health, mostly from high-income countries, has focused on depression in parents and its association with enduring consequences for educational attainment, social functioning and future productivity, consequences that raise concerns for affected families and for broader society.^2^^–^^4^ At the population level, these intergenerational pathways contribute to the accumulation of disadvantage and losses in human capital, particularly when multiple risks exist in contexts of socioeconomic adversity.^5^

However, vulnerability during the transition to parenthood is not evenly distributed. The risk of mental health difficulties is rooted in structural and social determinants that shape families’ capacity to cope, including poverty and economic insecurity; constrained educational opportunities; precarious employment; food and housing instability; and limited access to affordable and culturally responsive health and social services.^5^ Social environments further intensify risk. For example, social isolation, fragile support networks, experiences of discrimination and exclusion, exposure to intimate partner violence and the cumulative pressures of caregiving in underresourced settings can erode parental well-being and restrict pathways to care.^5^ These determinants often converge and reinforce one another, laying the groundwork for persistent but avoidable inequities in parental mental health within and across countries.^2^^,^^3^^,^^5^ In such contexts, exposure to intersecting structural and social stressors during pregnancy and the early years of childrearing places some parents at heightened risk of poor mental health outcomes, spanning both elevated psychological distress and constrained capacity for positive mental health.^2^^,^^3^

Positive mental health in parents is a vital yet underexplored frontier in global mental health research. Mental health promotion must extend beyond preventing illness to fostering emotional and social thriving in parents, who are the bedrock of child development and family functioning. This focus aligns with the World Health Organization’s (WHO) *Achieving well-being: a global framework for integrating well-being into public health utilizing a health promotion approach*,^6^ which calls for a whole-of-government and whole-of-society approach to embed physical, mental and social well-being across all policies and programmes, and emphasizes self-care, empowerment and resilience as central to health promotion. While traditional approaches often prioritize the treatment of psychopathology, emerging evidence highlights the transformative potential of cultivating psychological strengths in mothers, fathers and caregivers of young children. For example, the Nurturing Care Framework from preconception to adolescence,^5^ adapted from the WHO, United Nations Children’s Fund and World Bank *Nurturing care framework for early childhood development: a framework for helping children survive and thrive to transform health and human potential,*^7^ is a dynamic framework that is responsive to mechanisms that mitigate adversities, enhance resilience and promote the well-being of families, particularly those from marginalized groups.^5^

Positive mental health refers not merely to the absence of psychological illness, but to a dynamic state of emotional resilience, social connectedness, purpose and effective coping.^1^ Culturally grounded resources that strengthen meaning, belonging and coping can support positive mental health.^8^ Flourishing parents, as opposed to those experiencing chronic stress, poor mental health or limited emotional resources, are more likely to foster nurturing environments, engage in responsive caregiving and model adaptive behaviours that benefit child development.^1^ We therefore call for the systematic integration of strengths-based approaches into parenting and family mental health programmes, shifting from a sole focus on deficits to recognizing individual and community strengths and resources, particularly in settings where deficit-based narratives have overshadowed caregivers’ resilience and potential for growth. One promising approach to follow is the Triple P,^9^ a positive parenting programme that incorporates elements of positive mental health to build parental confidence and emotional well-being, and in so doing effectively improves both child and parent outcomes in the short and long term. The programme has successfully been implemented in 13 high-income and upper-middle income countries, suggesting a need to assess its effectiveness in lower-income settings. 

Historically, mental health research, whether focused on parents or the general adult population, has primarily focused on diagnosing and treating negative mental health states, inadvertently overlooking the considerable benefits gained when individuals flourish.^1^^,^^3^ This narrow paradigm limits our understanding of how flourishing can enhance psychological functioning, adaptability and life quality. A review of mental health research in Africa highlighted the limitations of dominant biomedical models, which often marginalize the mental health needs of non-clinical populations.^10^

Here we advocate for adopting frameworks such as the dual-continuum model, which clearly distinguishes mental illness from mental health and acknowledges that flourishing and distress can coexist.^11^ This dual-continuum model also has methodological implications, highlighting the need for mental health assessments that capture both clinical symptoms and indicators of positive functioning, such as validated flourishing or well-being scales, in parental mental health studies. The proposition that flourishing and distress can coexist in the same individual challenges prevalent conceptualizations of mental illness and well-being. On the one hand, an individual can be free from mental illness yet languish due to a lack of positive mental health. Conversely, someone diagnosed with a mental disorder can flourish if they have robust psychological and emotional resources. For parents, this distinction is crucial, underscoring the need for interventions targeting positive states such as positive expectation, emotional vitality and life satisfaction. Despite growing recognition of the importance of positive parental mental health, implementation remains uneven due to challenges related to measurement, service capacity, cultural relevance and the need to address broader structural constraints.^1^^,^^8^

Interventions that build psychological strengths and emotional resilience, particularly among vulnerable parents, show promise in enhancing positive mental health outcomes.^1^ Yet most existing programmes remain narrowly focused on the prevention or treatment of mental illness, with modest and inconsistent effects on long-term parental well-being and child development.^4^^,^^12^ A pressing need exists for rigorous research to design, implement and evaluate interventions that deliberately target flourishing and positive psychological states among parents. Such efforts should draw on emerging evidence linking parental well-being to child outcomes across diverse cultural and socioeconomic contexts.^1^ To be effective, such interventions must adopt an ecological perspective, addressing intrapersonal factors as well as family, community and structural supports that enable parents to thrive.^5^

Promoting positive parental mental health is a matter of individual well-being as well as a societal imperative with far-reaching implications for intergenerational health and resilience.^1^ Recognizing the central role that parents play in shaping family and community functioning can help strengthen support systems, protect future generations and foster thriving societies. Furthermore, integrating positive parental mental health into national health promotion agendas including programmes for adolescents and young adults can yield early and long-term benefits throughout the lifespan. Addressing intersecting factors such as gender, socioeconomic status and cultural background can facilitate the integration of positive mental health into young adult programmes across various initiatives.

Mental health professionals, educators, researchers, policy-makers and community leaders must jointly prioritize strategies that enhance psychological resources among parents. Programmes that cultivate optimism, self-efficacy and emotional regulation can help parents build psychologically adaptive family environments capable of withstanding adversity. A holistic and inclusive mental health agenda rooted in prevention, mental health promotion and equity can empower families and contribute to sustainable well-being at the population level. Embedding positive mental health components into national parenting programmes, primary health care and early childhood policies would help institutionalize these priorities and scale impact.

Expanding our understanding of positive mental health in parents and integrating it into policy and community practices has the potential to foster healthier, more resilient families and stronger communities, contributing to a sustainable future for society. Centring the well-being of parents in global mental health agendas would lay the foundation for sustainable development rooted in emotional, relational and intergenerational flourishing. This agenda also aligns with the sustainable development goals (SDGs), particularly SDG 3, that is, ensure healthy lives and promote well-being for all at all ages; and SDG 5, that is, achieve gender equality and empower all women and girls, which recognize the importance of mental health and caregiving equity in achieving inclusive societal progress.
